# Cost Implications from an Employer Perspective of a Workplace Intervention for Carer-Employees during the COVID-19 Pandemic

**DOI:** 10.3390/ijerph19042194

**Published:** 2022-02-15

**Authors:** Regina Ding, Amiram Gafni, Allison Williams

**Affiliations:** 1 School of Earth Environment and Society, McMaster University, Hamilton, ON L8S 4L8, Canada; awill@mcmaster.ca; 2 Centre for Health Economics and Policy Analysis, Department of Health Research Methods, Evidence and Impact (HEI), McMaster University, Hamilton, ON L8S 4L8, Canada; gafni@mcmaster.ca

**Keywords:** carer-employee, intervention, COVID-19, cost savings

## Abstract

In developed countries, population aging due to advances in living standards and healthcare infrastructure means that the care associated with chronic and degenerative diseases is becoming more prevalent across all facets of society—including the labour market. Informal caregiving, that is, care provision performed by friends and family, is expected to increase in the near future in Canada, with implications for workplaces. Absenteeism, presenteeism, work satisfaction and retention are known to be worse in employees who juggle the dual role of caregiving and paid employment, representing losses to workplaces’ bottom line. Recent discourse on addressing the needs of carer-employees (CEs) in the workplace have been centred around carer-friendly workplace policies. This paper aims to assess the potential cost implication of a carer-friendly workplace intervention implemented within a large-sized Canadian workplace. The goal of the intervention was to induce carer-friendly workplace culture change. A workplace-wide survey was circulated twice, prior to and after the intervention, capturing demographic variables, as well as absenteeism, presenteeism, turnover and impact on coworkers. Utilizing the pre-intervention timepoint as a baseline, we employed a cost implication analysis to quantify the immediate impact of the intervention from the employer’s perspective. We found that the intervention overall was not cost-saving, although there were some mixed effects regarding some costs, such as absenteeism. Non-tangible benefits, such as changes to employee morale, satisfaction with supervisor, job satisfaction and work culture, were not monetarily quantified within this analysis; hence, we consider it to be a conservative analysis.

## 1. Introduction

Aging populations are seen as a robust indicator for advanced living conditions in developed countries, and are often associated with high life expectancy, advanced healthcare and quality of life. Globally, older adults (65 years+) are the fastest-growing age cohort, growing at 3% per year [1]. In Canada, older adults comprise approximately 18% of the general population, with this proportion expected to increase in the near future [2]. This demographic trend warns of incoming pressures to labour markets and healthcare systems, as the comparatively smaller number of economically active Canadians are becoming more involved in the provision of care for the growing older adult cohort.

The implications of population aging are numerous and pervasive across different societal dimensions. Widespread population aging places strain not only on healthcare systems, which are not equipped to provide long-term care for chronic and age-related diseases, but also the family members charged with the administration of care. In this way, eldercare costs are two-fold: (1) approximately half of Canada’s total healthcare spending is on older adults, despite them comprising of about a fifth of the total population [3] and; (2) caregiving, performed by family and friends, is time-consuming and labour-intensive, with consequences for the carers [3,4].

In Canada, the neoliberalist reforms of the 1980s moved the bulk of eldercare provision away from the responsibility of the state and into the community and homes of the infirm; consequently, the majority of care (75%) is now provided by family members in the community, and often within private dwellings [5,6,7]. Long-term care homes, private community or homecare services with personal support workers and nurses, as well as mobility and hearing devices for the elderly, are costly and, as a result, usually not publicly funded. By outsourcing chronic and acute care provision to family and friends, this cost-effective solution frees up hospital beds, shortens hospital stays and releases healthcare resources to be utilized elsewhere [8]. Informal or family carers frequently opt to take on the responsibilities of a (health)care provider themselves, in lieu of hiring assistance. From a societal perspective, carers avert approximately CAD 25 billion in healthcare costs by performing labour themselves [9].

As of 2018, there are approximately 7.8 million carers in Canada aged 15 years or older; this represents approximately 25% of the total population [10]. The majority of care takes place within the care recipients’ independent dwellings as, in recent years, an increasingly geographically mobile population means that cohabitation is becoming less common [8,11]. Generally, carers tend to be women (54%), who are more likely to perform more time-intensive care tasks, such as physical or medical assistance. However, demographic trends suggest that care provision is becoming more equal among the genders, as men’s involvement in care has greatly increased. While men comprised just 23% of the carers in 2002, this figure has increased to 46% in 2018 [11,12]. In addition, most carers (44%) are between 45 and 65 in age, with the majority (47%) providing care for an elderly parent/parent-in-law [11]. Our paper herein spotlights eldercare situations as the most common care relationship. However, that is not to say that other types of caregiving are not present; for example, cancer is the second most common condition requiring care, followed by cardiovascular disease and mental illness. Developmental and physical disabilities each make up under 5% of all care situations [11].

The majority of Canadian carers (64%) report spending less than 10 h per week providing care on average; they perform tasks such as transportation, medical services, emotional support, financial assistance, housework and meal preparation [10]. However, care intensity is largely dependent on the personal situation and the needs of the care recipient, and is subject to rapid change [10]. Approximately one in five Canadian carers (21%) give what is considered to be high-intensity care provision, reporting over 20 h of weekly care per week [8]. Overall, the average annual time spent caregiving is estimated to be 290 h for the typical carer; however, it is projected that this figure will grow to 415 annual hours by 2050 due to the aging of the baby boomer cohort, coupled with increases in lifespan and smaller households with fewer children to provide care [7].

Carers themselves are increasingly regarded as a cohort that needs care. The unpaid labour performed by carers places considerable pressure on other aspects of their lives, which is reflected in reported adverse effects, such as increased stress, anxiety and depression, when compared to non-carers [13]. Carers may reduce social engagements, neglect personal responsibilities and reduce labour force engagement (in the form of shifting to part-time work or declining career advancement) in order to dedicate more time to caregiving. In other care situations, carers may feel unable to reduce their paid work hours due to the financial pressures of caregiving: approximately 41% of Canadian carers spend CAD 100–300 monthly on out-of-pocket caregiving-related expenses [4]. This situation leaves carers trapped in a precarious position and, thereby, more prone to burnout. Moreover, 28% of all carers are “sandwiched” between childcare and eldercare, introducing additional role tensions [11]. Although not a focal point within this paper, the sandwich carer demographic is a growing trend as dual earner families become more common, and women elect to have children later in life [14,15]. The process of care provision is emotionally charged and can sometimes be non-linear in its progression; as a result, carers often experience mixed emotions towards the carer role, ranging from affection to guilt, and sometimes resentment, as their care responsibilities interfere with their other commitments, such as personal time or work obligations [16,17,18].

From a labour market perspective, carers have the potential to create adverse disruptions to employers and firms if unsupported. There are approximately 6.1 million carer-employees (CEs) in Canada, defined as unpaid or family carers that are simultaneously engaged paid employment [11]. The common age range of carers (45–65 years) represents the most experienced workers in the labour force, with industry-specific skillsets and knowledge [11]. CEs’ impact on the Canadian labour market is substantial; it is estimated that an annual CAD 1.3 billion worth of productive work is lost due to care–work conflicts that result in CE absenteeism or turnover [19]. In the US, this estimate reaches USD 33.6 billion worth of annual lost productive work for all full-time CEs [20]. American employers may expect to lose approximately USD 2110 annually per CE, while in Canada, the estimate is CAD 8674 per CE lost annually [20,21]. Further, care–work conflicts can also result in adverse work outcomes that are not easily monetized, such as declines in mental health, poor employee morale and reduced job satisfaction [13,22]. Consequently, it is in the best interests of employers to mitigate these effects by supporting their CE population.

The COVID-19 pandemic has exposed the cracks in Canada’s problematic eldercare system, accelerating the downstream ramifications for employers. The pandemic has created dangerous conditions for current carers, produced new carers and has led to the acute exacerbation of CE burden. A report from the UK finds that not only are carers providing more care hours now than prior to the pandemic, but about 20% of CEs reported either reducing their work hours or leaving their job due to caregiving [23]. Overall, the ongoing global events of the past two years have demonstrated that the needs of CEs can and should no longer be ignored at an organizational level, if not for the sake of human decency, then for the impacts to the organization’s bottom line.

This manuscript is part of a multi-project research program on carer-employees and organizational policy. The objective of our overall research program is to introduce and evaluate the immediate impacts of a carer intervention within a workplace setting. Based on the Canadian Standards Associations’ *Carer-Inclusive and Accommodating Organizations Standard*, called the *carer standard* going forward, the intervention was customized for the specific needs of our partnered workplace [24]. This paper, as part of the larger program of research, specifically focuses on the cost implications of the intervention in order to build evidence for employer uptake of carer-related work initiatives. Through this, we hope to incentivize employers to implement mutually beneficial carer-supportive campaigns within the workplace. Although we seek to assess whether the intervention is cost-saving for the employer in a 6-month observational period (that is, if there is a net reduction in employer expenditure within our observed variables), we recognize that cost savings are only one of many considerations for a worthwhile intervention. Moreover, the short observation timeframe excludes future averted costs from our analysis.

## 2. Materials and Methods

### 2.1. Study Design

This study’s methods are based on a previous methodology created by Mofadi et al. (2019) under a prior research program [21]. This current project is a subsequent iteration of this original study, where an educational intervention has been implemented, albeit at a different workplace. Using cross-sectional surveys, we use a pre–post study design to observe and monetize changes in self-reported work variables (absenteeism, presenteeism, turnover, impact on coworkers) prior to and after the intervention. The participating workplace is the Canadian division of a large-sized multi-national engineering firm in the oil and gas industry, employing 3500 workers in Canada. A cost implication analysis was employed in order to evaluate if the intervention has been cost-saving from the employer’s perspective in the workplace studied. Specifically, we ask: (1) Does the intervention reduce costs (sometimes referred to as averted costs) from the employer’s perspective? (2) Do these averted costs outweigh the implementation costs of the intervention?

An educational intervention was designed and tailored to the needs of the participating workplace. A needs analysis (needs assessment and gap analysis) was conducted at the participating workplace in the summer of 2020 with the assistance of the employer’s Human Resources (HR) department. Key stakeholders and carer-employees were interviewed, in addition to implementing a preliminary version of a workplace-wide survey, which was circulated to all employees of the workplace, caregiving or otherwise. Both the interviews and survey assessed workplace culture, satisfaction with management, COVID-19 precautions and thoughts on the *carer standard*. A steering committee, composed of carer-employees and HR personnel, was formed in the fall of 2020 in order to guide the analysis and collaboratively design relevant and feasible intervention tools to best fit the participating workplace’s specific needs and gaps. A lack of a consistent and supportive workplace culture around carers was identified as a core issue to be addressed by the intervention.

In the first few months of 2021, the intervention was implemented over a 10-week period with the partnered workplace, in collaboration with the HR department. The goal of the intervention was to increase awareness and supports for carer-employees (CEs) at the workplace and, in doing so, to induce carer-friendly workplace culture change. Intervention tools included: standardized manager training, workplace “lunch and learns”, promotional posters, informational documents containing information on Federal and provincial carer resources, burnout management and recognition techniques and manager guidelines for communicating with carer-employees. Intervention content included: statistics, current research, recommendations and stories pertaining to carer-employees, with the overall goal of highlighting not only the struggles and needs of this growing population, but the role of the workplace in supporting their carer-employees. The overall objective of the intervention was to foster a more carer-friendly workplace, with appropriate resources and with compassionate and understanding managers, so that carer-employee work outcomes may be improved, which would, ultimately, lead to savings to the employer’s bottom line.

The intervention was designed as a one-time intervention, with an observational window of 6 months between pre-test and post-test. As a result, discounting was not applied.

### 2.2. Input Data

A workplace-wide survey was distributed immediately prior to the intervention (pre-test) as well 4 weeks following the intervention (post-test), capturing both demographic variables as well as work-related variables, such as absenteeism, presenteeism and turnover intention. The inclusion criteria for the surveys only required survey respondents to be employees (either full time or part time) of the participating workplace, with external contractors or consultants excluded. While we collected survey data from both CEs and non-carer employees, in this paper, we focus our analysis on CE data only. Non-carer employee data are only used as a baseline for the presenteeism and turnover variable, as described later. All analysis and calculations were performed in Excel.

We estimated the total number of CEs within the workplace via multiple scenarios, with our default scenario obtained by assuming that one-third of all employees were providing unpaid caregiving, based on current Canadian estimates [19]. A sensitivity analysis was also conducted to generate different scenarios with differing estimates of organizational CE proportions from the baseline of CEs making up one-third of all employees (from −10% to +10%).

From HR data, the hourly total compensation rates were calculated to be CAD 51.61 with benefits included (approximately 20% of the base wage). This wage rate was used to estimate the monetary value of time and labour of all employee groups, including CEs, supervisors and HR personnel. Where data were not available, we draw estimates of costs from the literature, as outlined in the following sections.

### 2.3. Intervention Costs

The intervention was assumed as a one-time cost, with costs derived only from the time and labour costs of employees involved, given that the intervention was implemented entirely virtually because of the COVID-19 pandemic. Researcher time and labour was estimated using the workplace wage rate as a proxy, as the role of the researcher is replaced by an employee in other such workplace situations. The intervention design and implementation are divided into 4 stages:

1. Needs analysis → front-end meetings with HR, interviews with key stakeholders, baseline assessment of workplace environment via workplace-wide survey;
2. Intervention design → design and creation of intervention tools with an internal steering committee, based on the needs identified from the previous stage;
3. Intervention implementation → email advertisement of promotional posters, planning and execution of training and lunch and learns;
4. Monitoring and evaluation → post-intervention interviews and survey assessment.

Monetary valuation is based on 2021 Canadian dollars; no discounting was applied, as all data were collected and the evaluation was completed within one year (December 2020 to June 2021).

### 2.4. Costs Saved to Employer Stemming from the Intervention

Using cross-sectional survey data, pre-test and post-test data were collected on four variables: (1) absenteeism, (2) presenteeism, (3) turnover and (4) impact on colleagues. These were converted into monetary estimates, using the average hourly organizational wage rate of CAD 51.61. The following sections detail the calculation of costs for each of the four variables of interest. We assigned the pre-test timepoint as the baseline or “no intervention” scenario, representative of the cost implication of unpaid caregiving on the employer. All the following costs were calculated twice, once using pre-test baseline data and again with post-test data from the survey. These cost estimations are used in the cost implication analysis, detailed later.

We take care to clarify that this analysis is a conservative estimate, as our evaluation is a partial estimation of costs. For example, potential outcomes (including their potential monetary implications), such as changes in quality of life, job satisfaction and work stress, are not captured.

#### 2.4.1. Absenteeism

Absenteeism, referring to lost productive time, was captured via the workplace survey using the human capital approach. We categorize absenteeism into two distinct types: short term (occasional single or partial days missed), and long term (extended leaves or reduction in work schedules/responsibilities). To estimate short-term absenteeism, CEs were probed about their absences (either full day or part day in the past 12 months) attributable to their caregiving responsibilities. Long-term absenteeism was queried by asking CEs if they had: reduced their weekly work hours for caregiving-related reasons and, if so, by how much, and how long this period of reduced work lasted. Using a conservative estimate, for CEs who were still on reduced working hours at the time of the survey, their long-term absenteeism endpoint was set as the day the participant responded to the survey.

Each workday was assumed as the standard 8 h, with the monetary conversion of this time estimated with the workplace average wage of CAD 51.61 hourly. From the survey, a sample mean and an associated cost were generated and designated as the per-case cost. It should be noted that not all CEs in our sample reported either short-term or long-term absenteeism; given this, the per-case estimate refers to the costs associated with a CE that does report absenteeism. A separate per CE column averages the cost of absenteeism across all CEs in the sample.

#### 2.4.2. Presenteeism

Non-specific presenteeism, defined as reduced productivity at work for any potential reason, was assessed and scored using the World Health Organization’s HPQ questionnaire for all survey respondents pre-test and post-test, caregiving or otherwise. Respondents self-reported their work performance over the preceding 4 weeks. The maximum score of 10 indicated no lost work, and a lower limit of 0 indicated total lost work. This self-assessment was converted to a score out of 100 to signify the percentage of actual work performed and, by extension, the lost work. A sample mean for presenteeism was calculated for both carers and non-carers, with the non-carer presenteeism assumed as the baseline. Presenteeism scores were multiplied by the hourly wage rate and then by the total hours worked in a year (assumed at 2080 h, based on a standard 52-week work year), generating an estimate for the monetary cost of lost productivity.

Presenteeism score = 100 − (Participant self-reported productivity × 10)

#### 2.4.3. Turnover

Carers and non-carers were surveyed for their turnover intention by probing if they had considered quitting their job in the last 12 months. Non-carer frequency of responding “yes” was used as baseline for comparison. Turnover intention is frequently used as a predictor and, at times, a direct proxy for turnover behaviour, with the literature finding varying degrees of association between turnover intention and actual turnover in private industry. While not completely interchangeable, correlations ranging from 0.32 [25] to 0.45 [26,27,28] suggest moderate associations between the two variables. Our study, adopting a conservative approach, uses a 30% estimator for turnover behaviour, where 30% of employees that indicate turnover intention actually follow through. From this, we assumed that the costs of turnover were 6 months’ worth of mean employee income to account for recruitment and retraining costs [20].

#### 2.4.4. Impact on Colleagues

Deviation from normal work routines and responsibilities have the potential to impact CEs’ immediate work team, such as supervisors or coworkers. Common accommodations/tasks, such as supervisory support and meetings, reallocation of work to coworkers and other general troubleshooting, represent lost productive work hours that may have been utilized for other work tasks. To estimate these costs, CEs were asked how many hours they spent per month arranging work-related accommodations with colleagues or supervisors for unpaid caregiving reasons. This value was converted to a yearly estimate, and then doubled to account for time spent on the supervisors’/coworkers’ end. The mean value was translated to a cost amount using hourly wages.

### 2.5. Cost Implication

Our evaluation seeks to determine if the workplace intervention is cost-saving in the short term by assessing the following questions:

1. Does the intervention avert costs (absenteeism, presenteeism, turnover and impact on coworkers) for the employer?
2. Do these averted costs outweigh the implementation costs of the intervention?

For each of the cost categories obtained from our survey sample (absenteeism, presenteeism, turnover and impact on coworkers), mean costs per CE were used to extrapolate a sum workplace-wide cost, using the assumption that a third of all employers were carers. Workplace-wide costs were accordingly evaluated from the employer’s perspective twice: in the no intervention scenario (pre-test), and in the post-intervention scenario (post-test) over a 6-month period. Costs savings refer to the difference in cost between the two scenarios, with the assumption that this difference did not change during our observational window. Pre-test data are assumed as the baseline for comparison. Averted costs refer to the reduction in cost (immediately) after the intervention. Future averted costs were not measured due to our short timeframe and, as such, our analysis is a conservative approach to cost savings. A cost implication analysis was performed by subtracting the implementation costs of the intervention from the averted costs.

## 3. Results

### 3.1. Participant Demographics

The demographic breakdown of survey respondents is presented in Table 1. The workplace-wide survey was distributed twice, first in December 2020 (pre-test), and later in June 2021 (post-test). Participation in the survey was open to all employees of the workplace, with 44 CEs (45.3% of all responses) participating in the pre-test, and 40 (25% of all responses) participating in the post-test. Table 1 presents CE cross-sectional data only, given that CEs are our population of interest in this study.

Interestingly, the gender composition of our CE sample differed at both data collection points, ranging from male dominated (57.1% male) at pre-test to female dominated at post-test (62.5%). This is not necessarily reflective of the workplace’s overall makeup. As a male-dominated firm, approximately one-third of the overall workplace identifies as female, despite making up more than a third of our survey respondents. Overall, the 35–54 age group is most common during both time points; this is in line with the average age of the overall workplace, with a mean age of 43.4 for women and 45.9 for men. It should be noted that a number of participants at pre-test did refrain from identifying their gender and age, which limited further inferences being made on the gender and age of our sample. Demographic information, such as marital status, was not available for the overall workplace.

Of the 3500 total employees at the workplace, we generalized that one-third (N = 1155) were CEs based on the literature for CE prevalence across all industries in Canada. While we assume this as the default scenario, a sensitivity analysis was also conducted to examine differing levels of cost associated with CE prevalence above and below this baseline.

### 3.2. Intervention Costs

We estimate the total cost of the intervention to be CAD 21,056.88, based on labour estimates for the four cost categories outlined in the methods section. The largest costs are front- and back-loaded, with the needs analysis (CAD 7586.67) and the monitoring (CAD 7122.18) accounting for over half of all costs. Both of these cost categories made extensive use of researchers’ time, which was substituted with the mean workplace hourly wage. Table 2 below depicts the full cost breakdown.

### 3.3. Absenteeism

As depicted in Table 3, at the pre-test, approximately 45.5% of CEs in the sample reported having short-term absenteeism related to caregiving within the past 12 months, while only 16% report having taken long-term absences. From this sub-sample, mean short-term and long-term absenteeism is estimated to be 90.2 and 145.1 annual productive hours lost, respectively. This equates to an approximate cost of CAD 4655.22 and CAD 7490.68 in lost working hours to the employer for each CE that ends up having short-term or long-term absences, respectively. Generalizing to the entire workplace using the per-CE costs, at baseline or in a no intervention scenario, a combined CAD 3,820,237.41 is being lost annually due to caregiving-related absenteeism.

As outlined in Table 4, cost savings were observed post-test in short-term absenteeism (CAD—1020.33 per case) and long-term absenteeism (CAD—3706.11 per case), while increases in the cost from the baseline were found for CE-specific presenteeism (CAD +1610.23 per case). However, from the pre-test to the post-test, we observe an increase in the frequency of CEs reporting absenteeism, thereby offsetting some of the cost savings. In total, workplace-wide cost savings for absenteeism are approximately CAD 444,557.53.

Table 5 depicts the sensitivity analysis, outlining variations in costs employers may expect based on different organizational CE counts. The default scenario (one-third of total employees are CEs) is used throughout this paper.

### 3.4. Presenteeism

Using non-CE presenteeism rates as a baseline, we note that CE presenteeism is higher than non-CE rates at both pre- and post-test. At pre-test, the difference in CE and non-CE presenteeism costs are CAD 4508.65 per case; accordingly, the total cost of CE presenteeism to the workplace is estimated at CAD 5,207,490.29 in the no intervention scenario.

We observe that presenteeism for both CEs and non-CEs have increased at post-test, with the difference between CEs and non-CEs growing to CAD 6118.88 per case. In total, we estimate additional workplace-wide costs of CAD 7,067,308.25 for CE presenteeism.

### 3.5. Voluntary Turnover

Using a similar approach as with presenteeism, we assumed non-CE turnover intention as the baseline for both the pre- and post-test. Using the reported turnover rates in Table 6, together with the estimation that one-third of participants who indicated turnover intention would also embody turnover behaviour, we extrapolate that, across the entire workplace, the employer would expect an annual turnover of approximately 175 CEs and 330 non-carer-employees at pre-test (sum total of 505). This extrapolation is increased to 219 CEs and 358 non-carer-employees (577 total) at post-test. These turnover estimates comprise approximately 14.4% and 16.5% of the total workplace labour force at pre-test and post-test, respectively. This is roughly aligned with what we know from the literature to be the Canadian voluntary turnover estimates of 12% across all industries [29]. The difference in turnover rates between CEs and non-CEs was used to estimate how many extra CEs would leave the workforce when compared to non-CEs in an equal sample group. From this, we estimate that 12 extra CEs would turnover at pre-test compared to an extra 42 CEs at post-test.

Per case estimates of turnover costs are assumed to be 50% of annual salary for both CEs and non-CEs. Workplace-wide voluntary turnover costs are estimated to be CAD 661,480.28 compared to non-CEs at pre-test. These costs increase to CAD 2,275,606.28 at post-test.

### 3.6. Impact on Colleagues

At pre-test, 50% of the CE sample reported having spent time communicating about, or making some form of work accommodation/arrangement (i.e., requesting telecommuting, rearranging work) with their supervisors or coworkers due to caregiving reasons. The mean number of hours spent on such tasks is estimated to be 104.2 h annually, accounting for CE time as well as supervisor/coworker time. We assessed that the per-case costs are approximately CAD 5377.76 for each employee that reports impacts on colleagues, resulting in a workplace cost of CAD 3,105,657.56.

We note increased time spent on communicating and arranging work at post-test, leading to an increase in costs to the employer (CAD +6325.73 per case, up from CAD +5377.76). Thus, workplace-wide costs are up to CAD 4,352,317.23 at post-test.

### 3.7. Net Cost Analysis

At the baseline pre-intervention scenario, we observe total caregiving costs to the employer to be CAD 12,794,865.53, which averages out to a per-CE cost of CAD 11,077.81. This is compared to a post-intervention workplace cost of CAD 17,070,911.64, or CAD 14,780.01 per CE. We observe negative cost savings, or increased costs of CAD 4,276,046.11 across the workplace. Intervention costs were found to be CAD 21,056.88. In sum, the net cost of the intervention, including the cost of the intervention and the negative averted costs, is CAD 4,293,594.19.

## 4. Discussion

In this study we attempt to identify the monetary implications of a workplace-based intervention for improving carer-employee (CE) outcomes in a large-sized engineering firm. In doing so, we hope to generate evidence of whether the intervention is capable of paying for itself in the short term via averted costs. We take care to highlight that we utilize a conservative approach, with a narrow range of costs as well as a short timeframe; as such, we do not acknowledge or monetize all forms of savings or future savings. As a result, this evaluation is only a conservative cost implication analysis from the employer’s perspective.

It is estimated that the total burden of caregiving on the employer at baseline (no intervention) is CAD 12,794,865.53. Overall, we did not observe the intervention being effective at averting costs. Our analysis found that the intervention did not pay for itself, but rather cost the workplace CAD 4,293,594.19 when compared to the baseline (inclusive of the CAD 21,056.88 cost of the intervention).

While we did not find the intervention to be cost-saving overall in the 6-month period, the intervention exhibited evidence of savings in some areas. When examining the cost items separately, from pre-test to post-test, we observed cost savings in absenteeism, both short-term and long-term. These savings amount to CAD 3,820,237.41 workplace-wide, or CAD 3,799,180.53 if factoring in intervention costs. These savings, however, are negated by increased expenses in other cost categories, such as presenteeism, turnover and impact on colleagues.

Although the intervention did not report overall cost savings, we take notice of several subsidiary findings of interest to employers. First, we note that turnover intention is higher among CEs than non-CEs. This finding aligns with results from the literature, which posits that CEs are more likely to turnover due to work–life conflicts [30]. This forecasts a concerning trend for workplaces: as CE prevalence is projected to increase in the near future, there may likely be a rise in turnover costs to employers.

Second, we would caution the interpretation of the high presenteeism costs. As with many workplaces, our partner workplace largely operated under a work-from-home mandate due to COVID-19. As a result, we anticipate that while reported presenteeism during working hours was high, this may not directly represent lost work. An earlier qualitative paper from this same workplace found that CEs were re-contracting their work hours outside of traditional working times, as remote working provides carers greater agency to negotiate their home, care and work responsibilities using a schedule that is most beneficial to them [31]. In this regard, we speculate that employees are still accomplishing the majority of their work tasks, albeit outside the usual 9–5 schedule due to work disruptions at home. This is supported by findings from the COVID-19 literature; a European study found high rates of presenteeism among staff at a university workplace due to stress associated with working from home and living conditions [32]. Similarly, remote working from home has long been associated with benefits, such as greater schedule control and flexibility, but is characterized as having an increase in distractions, even prior to the pandemic [33]. Consequently, it is possible that the CAD 7,067,308.25 cost estimate of CE presenteeism, which comprises a large proportion of the costs, may not accurately represent lost work.

The literature on carer-employee workplace interventions is sparse, and even fewer studies evaluate the cost implications of carer-employee interventions, let alone during the pandemic; this makes comparisons difficult. From our research group, a pilot evaluation at a similarly sized (4000 employee) university workplace established a baseline cost to the employer of CAD 8,916,342 (CAD 8674 per CE) under non-pandemic conditions [21]. Our present study calculated the baseline cost as CAD 12,794,865.53 (CAD 11,077.81 per CE), although we include the benefits paid by the employer in our study. In the previous pilot study, the majority of costs originated from absenteeism and turnover costs; in the current study, presenteeism is the largest contributor to costs. We theorize that this shift is largely a function of remote working mandates and burnout during COVID-19, increasing presenteeism rates. Our prior pilot study found that a similar workplace intervention was able to generate positive outcomes in work and health variables, such as family-supportive supervisor behaviour and self-reported health; however, absenteeism and presenteeism were unassessed in these studies [34,35]. Elsewhere in the literature, workplace interventions based on work–life policies in professional industries tend to produce increased productivity at an organizational level [36]. However, monetary estimates are lacking within the literature. Further, within the context of the pandemic, it is unclear how our estimates may differ from estimates obtained prior to the pandemic.

While this intervention was not found to be cost-saving in the short term, we acknowledge that there are several variables at play. The conservative approach may have excluded the evaluation of other tangible but unmeasured benefits, such as improvements in job satisfaction or supervisor behaviour. Indeed, a large focus of the intervention was centred on creating a carer-friendly workplace culture through establishing a supportive environment for CEs; this may not have necessarily translated into absenteeism, presenteeism, turnover and/or impact on coworker rates.

Further, due to the design of the intervention, it may be expected that increases in cost items, such as impact on coworkers, may develop to an extent. Given that the intervention was centred around employee supports with an emphasis on supervisory support/compassion, this may lead to more CEs seeking out these supports, thereby increasing costs in the form of greater supervisor time spent on caregiving issues. These increases in immediate costs may be beneficial if they avert greater costs in the future. In a similar realm, the novelty of the intervention and short timeframe of data collection may also mean that benefits related to averted costs may not be apparent yet. While the initial costs are front-loaded, benefits to carers may not manifest until several years after, potentially when situations characterized by higher care burden arise.

Finally, to address the elephant in the room, COVID-19 restrictions were in force during the entirety of our study, with data collection for pre-test and post-test occurring during Canada’s second and third waves of cases. During these waves, lockdowns and stay-at-home orders were enforced, with only essential services (i.e., groceries stores, pharmacies) remaining open, while schools and care services (i.e., respite care, personal support worker services) were closed. It is likely possible that these lockdowns may have prompted respondents to inflate reports of absenteeism, presenteeism, turnover and impact on coworkers. With COVID-19, it is difficult to ascertain if the intervention was truly ineffective, or if the global state of work rendered intervention effects undetectable. It may also be that information-based interventions are less effective during global crisis conditions, due to underlying anxieties. Gabriel and Aguinis recommend targeted workplace interventions for burnout during the pandemic (i.e., mindfulness meditation and cognitive behaviour therapy), as these types of interventions are more likely to mitigate emotional exhaustion and distress tolerance [37].

One of the main strengths of the intervention is that it is one of the first of its kind to introduce a workplace intervention for carers during the COVID-19 pandemic. While the pandemic did complicate the design and implementation of the intervention, particularly as the intervention had to be executed and monitored remotely, the need for such an intervention at workplaces was salient and timely. Given that the COVID-19 virus impacted the elderly and immunocompromised most severely, the importance of caregiving was illuminated on a larger scale within mainstream media [38,39]. At the same time, the restructuring of work meant that employers and employees were questioning the dominant paradigm and the future of work [40,41]. In combination, the pandemic’s “silver lining” was that it facilitated discussions of workplace programs and policies to support CEs, who were being disproportionately burdened by the pandemic. The workplace should be recognized as an agent with the potential for the facilitation of supports for all employees, carers included. This is crucial, as shifts in the social structure of labour due to women continuing to enter the workforce mean that the division of labour becomes more equitable in households, and work–life conflicts become more common across all employees [42]. Pandemic impacts on the work landscape, and the associated sequelae, may be rampant for years to come, during which changes in work structure and responsibilities may become normalized. From a policy perspective, it may even be preferable to “strike while the iron is hot”: disaster management literature posits that disasters are often transformative agents, opening the door for policy change vis-à-vis consciousness raising and pressure for change [43].

While we did not find immediate cost savings within the timeline of our study (6 months), this does not preclude the possibility of future savings. As such, it is difficult to conclusively determine that the intervention is not cost-saving overall; rather, it is not cost-saving in the short term.

In fact, potential future benefits to the employer include: (1) the organizational framework has been established so that future iterations and modifications of the intervention are less time-intensive; and (2) organizational support for employee wellbeing endeavours communicates commitment to employees and is positively associated with employee loyalty [44,45]. Given that workplace culture change often takes place over the span of years, it is therefore unsurprising that there was a lack of immediate cost savings [46,47]. Even within our paper, we monetized only a slice of potential intervention effects. It may be possible that, in the future, cost savings may manifest in other areas that are not captured within the variables examined in this paper. One caveat that should be noted is that, within our present analysis, the intervention is a one-time cost. In the future, should the intervention be implemented again, costs would rise accordingly; however, we anticipate that these implementation costs may be lower due to the existing groundwork in place.

Given the global rate of aging, caregiving conflicts with work are predicted to escalate in the future as the number of carers continue to grow. Employers, HR professionals and policymakers should be proactive in implementing carer-supportive programs and policies not only from a social responsibility lens, but also as an incentive for employees. Given the unique context of COVID-19, we do not seek to generalize our findings to non-pandemic scenarios but, instead, position this as an in-depth examination of an intervention for carers customized for COVID-19, with the potential for long-standing effects. We do not anticipate that this will be the last pandemic or crisis that has workplace repercussions and, as a result, our findings may be useful for future events.

## 5. Policy and Program Implications

Within the field of economics, the concept of cost savings is used as a tool for decision making when budgets are limited and competing costs exist. Positive savings indicate that an intervention does not require additional resources and, thus, is easier to implement, as it does not compete with other programs for the scarce resources available. As a matter of fact, it releases resources to be used elsewhere. The cost savings of an intervention, however, are not the sole end goal of an intervention, nor are they mutually exclusive with intervention effectiveness. Non-cost-saving interventions become a matter of maximizing goals within an existing budget.

It is important to reiterate that, in the context of our study, the lack of observed intervention effects and cost savings does *not* necessarily mean that: (1) this will be the case in all scenarios, and (2) the *carer standard* intervention is not worth implementing. As previously noted, the conservative nature of our approach meant that not all costs (and subsequently averted costs) were captured and monetized in our analysis. It is possible that a more comprehensive analysis, which includes and monetizes additional variables and captures the impacts over an extended period of time, may find a different result.

Irrespective of whether our implemented intervention does pay for itself or not (in the short term), the issue of unsupported CEs in the workplace remains; employers need to consider CE interventions, such as the *carer standard*, in the workplace, if not for the sake of cost savings, then certainly out of respect for human dignity and those entrusted in the care and protection of it. Our intervention was found to be not immediately cost-saving; that is, it requires additional workplace resources. In this way, the goals of the intervention (i.e., workplace culture changes) are prioritized over cost savings. There will come a time where each one of us will be placed in the position of being a carer, and eventually a care recipient. Employers should utilize this cost implication analysis as part of, but not the sole criterion for, their decision making. Indeed, innovative and progressive campaigns are rarely cost-saving, and while this intervention was not found to pay for itself, it does not mean that it is unaffordable [48]. However, the lack of short-term cost savings does raise questions concerning where the resources that fund such an intervention come from, and what other programs are being rejected in its favour. Private companies need to determine their own organizational priorities, and their acceptable timeline on returns on investments. We propose that employers, policymakers and all relevant stakeholders, as part of their individual decision making, consider the current and future needs of their labour force and view CE interventions as an investment in their CEs, and not merely a cost-saving tool.

## 6. Limitations

We acknowledge that while the timing of the COVID-19 pandemic provided benefits, it also introduced several limitations. Firstly, given the restrictions on “regular” work (i.e., remote work or working from home), it is difficult to generalize findings outside of a pandemic scenario, given that the measured variables were reflective of participants’ pandemic work situation. For example, absenteeism rates may be lower during the pandemic compared to non-pandemic situations due to the prevalence of remote working and the movement of many services (i.e., medical appointments) towards a digital medium. The evaluation of the intervention is difficult during the pandemic, as it is not wholly possible to isolate intervention effects from provincial and national events associated with the crisis. Further, given that the impacts of the carer-friendly workplace culture change intervention may not have been fully experienced by the time of the post-intervention survey, the full impact of the intervention may not have been comprehensively captured.

Another limitation was the small sample size used for analysis. Despite being a large-sized workplace, we were only able to recruit 44 and 40 CEs at the pre-test and post-test, respectively, making it difficult to determine if this sample is representative of the entire CE cohort at this workplace. In addition to the known challenges of recruiting CEs to research, which were further complicated by the pandemic, post hoc discussions with HR endorsed that the recruitment difficulties were likely attributable to the influx of email communication associated with remote working, leading to emails regarding the surveys being drowned out [49]. While we treat CEs as a single group, we must also recognize that there is immense diversity within the carer identity. For example, sandwich carers are a sub-group of carers with unique and exacerbated role tensions due to the intersection of childcare, eldercare and work responsibilities. We do not examine the specific needs of this group in this paper and, as such, we are limited in establishing the demographic context.

Lastly, the cross-sectional design of the study poses limitations in interpretation. While our pre-test and post-test sample remained somewhat consistent in makeup, it is not possible to conclusively determine cause and effect with respect to the intervention and the observed outcomes.

## 7. Conclusions

Global aging has underscored the need for workplaces to be supportive of their carer-employees, with this being exacerbated during the COVID-19 pandemic. This study examines the cost implications of a workplace intervention, the implementation of the *carer standard*, targeted at creating a carer-supportive work culture. Using a conservative approach, we observed and monetized costs relating to absenteeism, presenteeism, turnover and impact on coworkers prior to and after the implementation of the workplace intervention. We find that although the intervention did not pay for itself within a 6-month window (i.e., it was not cost-saving), being calculated at a net cost of CAD +4,293,594.19 across the workplace, pandemic conditions made it difficult to determine the true impact of the intervention. Nonetheless, the implementation and execution of such an intervention may provide non-tangible benefits to the employer, such as the establishment of groundwork and strong organizational messaging, paving the way for future iterative campaigns. Subsequent studies should seek to incorporate additional variables in the analysis, extend the study window in order to fully capture intervention impacts and examine workplaces in other industries, in order to explore future cost savings and the reliability and generalizability of the findings.

## Figures and Tables

**Table 1:** Demographic breakdown of respondents at pre-test and post-test.

Demographic		Pre-Test (*n* = 44)	Post-Test (*n* = 40)
Gender			
	Male	20 (45.5%)	15 (37.5%)
	Female	15 (34.1%)	25 (62.5%)
	Other	0 (0%)	0 (0%)
	Missing/NA *	9 (20%)	0 (0%)
Age			
	18–24	1 (2.3%)	2 (5%)
	25–34	4 (9.1%)	8 (20%)
	35–44	13 (29.5%)	8 (20%)
	45–54	11 (25%)	17 (42.5%)
	55–64	5 (11.4%)	5 (12.5%)
	65+	2 (4.5%)	0 (0%)
	Missing/NA *	8 (18.2%)	0 (0%)
Marital Status			
	Married/Common law	28 (63.6%)	29 (72.5%)
	Separated/Divorced	2 (4.5%)	3 (7.5%)
	Single	6 (13.6%)	7 (17.5%)
	Widowed	0 (0%)	1 (2.5%)
	Other	0 (0%)	0 (0%)
	Missing/NA *	8 (18.2%)	0 (0%)

* Missing/NA data reflect participants choosing not to answer demographic questions.

**Table 2:** Cost categories for the intervention, from initial needs analysis to the monitoring stage.

Intervention Cost Categories		Time (hours)	Cost
Needs Analysis	Front-end HR meetings	9	CAD 464.49
	Interview with key stakeholders	18	CAD 928.98
	Survey analysis	120	CAD 6193.20
Intervention Design	Design and creation of intervention tools	80	CAD 4128.80
	Feedback from steering committee	20	CAD 1032.20
Intervention Implementation	Circulation of promotional posters	3	CAD 154.83
	Planning and execution of lunch and learns/seminars	20	CAD 1032.20
Monitoring and Evaluation	Post-test interviews	18	CAD 928.98
	Survey analysis	120	CAD 6193.20
TOTAL		408	CAD 21,056.88

**Table 3:** Mean absenteeism (in hours) in pre-test and post-test.

	Pre-Test Lost Working Hours (Past Year) (N = 44)			Post-Test Lost Working Hours (Past Year) (N = 40)		
Lost Time	Mean	SD	CEs Percent *	Mean	SD	CEs Percent *
Long term	145.14	176.1	15.91%	73.33	76.73	22.00%
Short term	90.2	130	45.45%	70.43	101.92	57.50%
Impact on coworkers	104.2	101.4	50.00%	122.57	126.024	59.57%

* CE percent denotes the percentage of the CE sample that reported each form of lost time.

**Table 4:** Cost breakdown in each cost category at pre-test compared to post-test.

	Pre-Test (No Intervention)			Post-Test (Post-Intervention)		
Cost Item (Annual)	All CE Cost	Per-Case Cost	Per-CE Cost *	All CE Cost	Per-Case Cost	Per-CE Cost *
Short-term absenteeism	CAD 2,443,747.15	CAD 4655.22	CAD 2115.80	CAD 2,414,022.85	CAD 3634.89	CAD 2090.06
Long-term absenteeism	CAD 1,376,490.26	CAD 7490.68	CAD 1191.77	CAD 961,657.03	CAD 3784.56	CAD 832.60
Presenteeism Non-CE	NA	CAD 21,136.98	NA	NA	CAD 22,435.90	NA
Presenteeism CE	CAD 29,620,700.71	CAD 25,645.63	CAD 25,645.63	CAD 32,980,771.82	CAD 28,554.78	CAD 28,554.78
Difference in presenteeism	CAD 5,207,490.29	CAD 4508.65	CAD 4508.65	CAD 7,067,308.25	CAD 6118.88	CAD 6118.88
CE Turnover	CAD 661,480.28	NA	CAD 572.71	CAD 2,275,606.28	NA	CAD 1970.22
Impact on colleagues	CAD 3,105,657.56	CAD 5377.76	CAD 2688.88	CAD 4,352,317.23	CAD 6325.73	CAD 3768.24
TOTAL	CAD 12,794,865.53	CAD 43,169.29	CAD 11,077.81	CAD 17,070,911.64	CAD 42,299.97	CAD 14,780.01

* Per-CE Cost columns refer to the typical cost associated with one CE, and is calculated using total workplace cost and CE organizational count. The Per-Case Cost columns refer to the costs associated with a single instance of (mean) absenteeism, presenteeism and impact on colleagues, recognizing that not all CEs may experience absenteeism, presenteeism, etc.

**Table 5:** Sensitivity analysis of various estimations of CE count (total number of CEs employed at the workplace).

Scenario	CE Count Across Workplace	All CE Cost Pre-Intervention	All CE Cost Post-Intervention	Net Difference *
CE Count Increased by 10%	1143.45	CAD 14,074,352.09	CAD 18,778,002.80	CAD 4,703,650.72
CE Count Increased by 5%	1091.475	CAD 13,434,608.81	CAD 17,924,457.22	CAD 4,489,848.41
Default	1155	CAD 12,794,865.53	CAD 17,070,911.64	CAD 4,276,046.11
CE Count Decreased by 5%	987.525	CAD 12,155,122.26	CAD 16,217,366.06	CAD 4,062,243.80
CE Count Decreased by 10%	935.55	CAD 11,515,378.98	CAD 15,363,820.48	CAD 3,848,441.50

* Net difference does not include the cost of the intervention.

**Table 6:** Turnover intention of respondents at pre-test and post-test.

	Have You Considered Leaving Your Job in the Last 12 Months?		
		Yes	No
Pre-Test	Carer	45.45%	54.55%
	Non-Carer	42.25%	57.75%
	Difference	3.20%	−3.20%
Post-Test	Carer	56.76%	43.24%
	Non-Carer	45.74%	54.26%
	Difference	11.01%	−11.01%

## Data Availability

Restrictions apply to the availability of data. Data were obtained with the assistance of the HR department in our participating workplace. Data may be available on request from the corresponding author, with permission from the participating workplace.
